# Race is not a factor in overall survival in patients with triple negative breast cancer: a retrospective review

**DOI:** 10.1186/2193-1801-2-516

**Published:** 2013-10-07

**Authors:** Athena Starlard-Davenport, Katherine Glover-Collins, Issam Mahkoul, Laura Hutchins, Kent Westbrook, Soheila Korourian, Kimberly Enoch, Michael Preston, Shakia N Jackson, V Suzanne Klimberg, Ronda Henry-Tillman

**Affiliations:** Department of Cancer Control and Population Sciences, University of Arkansas for Medical Sciences, Little Rock, AR 72205 USA; Division of Breast Surgical Oncology, University of Arkansas for Medical Sciences, Little Rock, AR 72205 USA; Department of Medicine, Division of Hematology/Oncology, University of Arkansas for Medical Sciences, Little Rock, AR 72205 USA; Department of Pathology, University of Arkansas for Medical Sciences, Little Rock, AR 72205 USA; Cancer Control, Cancer Institute, Ladies’ Oncology Clinic, Winthrop P. Rockefeller Cancer Institute, University of Arkansas for Medical Sciences, Little Rock, AR 72205 USA

**Keywords:** Triple negative breast cancer, Race, Overall survival

## Abstract

The purpose of this study was to determine if race is a factor on overall survival when stage at diagnosis is compared. In this study, a total of 93 women with triple negative breast cancer (TNBC) were evaluated for survival outcomes after diagnosis between the year 2000 through 2010. Thirty-five patients (38%) were African American (AA), and 58 patients (62%) were Caucasian. Overall survival rates were estimated using the Kaplan-Meier method and compared between groups using the log-rank test. Student’s *t*-test was used to calculate differences in cancer recurrence and mortality rates by stage and race. Cox proportional hazards ratios were used to determine the association of patient and variables with clinical outcome. Of women diagnosed with stage 1 breast cancer, the overall survival rates for AAs was 100% compared to Caucasians at 94% (95% CI, 0.003 to 19; *P* = 0.5). For women with stage 2 breast cancer, overall survival for AA women was 85% and for Caucasian women was 86% (HR = 0.8; 95% CI, 0.3 to 2.6; *P* = 0.73). For advanced stages (stage 3 and 4), survival for AA women were 78% and 40% for Caucasian women (HR = 0.6; 95% CI 0.2 to 1.98; *P* = 0.43). Rates of recurrence and mortality were not significantly different between AA and Caucasian TNBC patients. After controlling for patient variables, race was not significantly associated with OS (HR = 1.24; 95% CI, 0.32 to 5.08; *P* = 0.74) when comparing AA to Caucasian patients. Our study suggests that race does not have an effect on overall survival in African American and Caucasian women diagnosed with TNBC in Arkansas.

## Introduction

Breast cancer is a major health problem that is expected to affect more than 230,000 women in the United States in 2011 (Brawley & Berger 2011). Fortunately, for Arkansas, it was reported that there was a 9.3% decline in the incidence of invasive female breast cancer in 2003, and the decline continued through 2005 (Balamurugan et al. 2009). This decline in breast cancer incidence was significantly higher (p < 0.05) among invasive cancers, particularly among women ages 50 and older and for those having estrogen-receptor positive tumors (Balamurugan et al. 2009). Although that report is encouraging, it has been reported that in Arkansas, death rates have increased by 1.9% per year among AA women since 1975; whereas, the death rate has declined by 1.6% per year since 1987 among Caucasian women (DeSantis et al. 2008). This finding is overwhelming in comparison to all other states, except Mississippi (DeSantis et al. 2008). These observed differences in death rates among African American (AA) women may be attributed to a variety of factors including advanced stage of disease at the time of diagnosis (Weir et al. 2003), less prompt access to medical care (Shavers & Brown 2002), and socioeconomic factors (Brawley 2002). Additionally, an aggressive form of breast cancer known as triple negative breast cancer (TNBC) may be a factor resulting in increased mortality rates among young, pre-menopausal AA women (Amirikia et al. 2011).

TNBC is largely characterized by tumors that do not express the estrogen receptor (ER), progesterone receptor (PR), or HER-2/neu receptor (Reis-Filho & Tutt 2008). The lack of receptors has made adequate therapies elusive (Hudis & Gianni 2011), as conventional chemotherapy and endocrine therapies are rendered ineffective due to lack of a therapeutic target. Epidemiological studies provide evidence of significantly higher mortality and prevalence rates due to TNBC among young, pre-menopausal AA women (Amirikia et al. 2011; Carey et al. 2006; Tammemagi 2007). However, there are limited studies that specifically address the question as to whether race independently is a prognostic factor in predicting survival outcomes in TNBC patients. Furthermore, to our knowledge, there are no reports in the published literature that assess triple negative or other types of breast cancer trends in Arkansan women as it relates to ethnicity, treatment response, cancer recurrence and overall clinical outcome. Therefore, this retrospective study was undertaken to evaluate the effect of race on survival outcomes among AA and Caucasian women diagnosed with TNBC from the year 2000 to 2010 in Arkansas.

## Materials and methods

### Study design

Institutional review board approval was obtained from the University of Arkansas for Medical Sciences (UAMS) prior to commencement of this retrospective study. The retrospectively maintained database in the Women’s Oncology Clinic of the Winthrop P. Rockefeller Cancer Institute of UAMS was queried from June 2000 to October 2010 to identify all patients with a diagnosis of stage I-IV biopsy-proven invasive TNBC who received neoadjuvant or adjuvant chemotherapy and/or radiotherapy. The final analysis included 93 patients. Patient variables recorded included race, menopausal status, tumor characteristics, stage and grade at diagnosis, presence of residual disease after treatment, recurrence information and treatment details including receipt or non-receipt of chemotherapy and/or radiotherapy. Race information was self-reported based on data derived from forms completed during the patient’s initial visit to the clinic.

### Staging and pathological assessment

The American Joint Committee on Cancer Criteria (sixth edition) (Singletary et al. 2003) was used to define the initial clinical and final pathologic stage of breast cancer patients. Histologic type and grade were defined according to the WHO classification system (The World Health Organization 1982) and modified Black’s nuclear grading system (Black & Speer 1957), respectively. Breast pathologists at UAMS reviewed all pathologic breast specimens and invasive carcinomas were confirmed on initial core biopsy specimens. In our study, we defined pathologic complete response (pCR) as the absence of invasive cancer in both the breast and axillary lymph nodes on final pathologic assessment. All surgical breast and axillary lymph node specimens were reviewed to identify the presence or absence of residual invasive and *in situ* disease.

Pathologic diagnosis and determination of hormone receptor status was determined using standard immunohistochemical methods. Tumors with less than 1% nuclear staining were considered to have a negative status for ER and/or PR. HER-2/neu receptor status was assessed by immunohistochemistry only if the results were 0/1+ or 3+ staining and by fluorescence *in situ* hybridization (FISH) confirmation if 2+ immunohistochemistry staining was present.

### Statistical analyses

Race was divided into the following two groups: AA and Caucasian female patients. Because of the small number of patients with grade 1 tumors, patients with grade 1 or 2 tumors were grouped together.

Patient’s race and tumor characteristics were compared across groups with the Chi-square test or Wilcoxon rank sum test, as appropriate. Overall survival (OS) was defined as the time from initiation of chemotherapy or surgery to date of death due to any cause or date of last follow-up. Recurrence free-survival (RFS) was measured from the date of definitive surgery to the date of first documented recurrence (local or distant) or date of last follow-up. Patients who died before experiencing a disease recurrence were censored at their date of death in the analysis of RFS. Survival outcomes were estimated using the Kaplan-Meier product-limit method and compared between groups using the log-rank statistic. Student’s *t*-test was used to calculate differences in cancer recurrence and mortality rates by stage and race. We fitted Cox proportional hazards models for each survival outcome to determine the simultaneous relationship of patient and tumor variables with each outcome. For each outcome, pCR and clinical stage achieved statistical significance by the likelihood ratio test and were included in the Cox model. All statistical analyses were performed using GraphPad Prism, version 4 software (La Jolla, CA). For all of the statistical tests, a two-sided *p*-value of less than 0.05 was considered statistically significant.

## Results

### Patient characteristics

Between June 2000 and October 2010, 93 patients diagnosed with stage I-IV TNBC and treated at UAMS in Little Rock, Arkansas were included in this report. Table 1 summarizes the characteristics of the 93 patients included in this report.

**Table 1:** **Patient characteristics by race**

	AA (n = 35)		Caucasian (n = 58)		
Characteristic	No. of patients	%	No. of patients	%	***P***
**Age, years (mean ± SD)**	50.9 ± 13.5		56.3 ± 13.6		.163
Minimum	26		30		
Median	50		53		
Maximum	76		82		
**Menopausal status**					.232
Premenopausal	17	48.6	21	36.2	
Postmenopausal	18	51.4	37	63.8	
**Histology**					.696
Ductal	33	94.3	50	86.2	
Other	2	3.45	8	13.8	
**Clinical Stage**					.564
I	7	20.0	16	27.6	
II	20	57.1	28	48.2	
III	4	11.4	14	24.1	
IV	5	14.3	1	1.72	
**Grade**					.685
I/II	0	0	8	13.8	
III	33	94.3	47	81.0	
Unknown	2	5.71	3	5.17	
**Neo/Adj chemotherapy**					.121
Yes	30	85.7	45	77.6	
No	3	8.57	10	17.2	
Unknown	2	5.71	3	5.17	
**Adjuvant radiotherapy**					.143
Yes	17	47.2	25	41.7	
No	19	52.8	35	58.3	

Of the 93 patients, 35 patients (38%) were AA, and 58 patients (62%) were Caucasian. Median age at diagnosis was 50 years (range, 26 to 76 years) among AA patients and 53 years (range, 30 to 82 years) among Caucasian patients. There was no significant association between menopausal status and race (*P* = 0.232). Overall clinical stage and grade at diagnosis were not significantly different between races (Stage: *P* = 0.564; Grade: *P* = 0.685). The majority of patients were diagnosed with clinical stage I/II (76%) TNBC and had grade 3 breast cancer (86%). Both stage and grade were similar between races. Invasive ductal carcinomas were the predominant histologic type of cancer among both AAs (94%) and Caucasian (86%) patients. Of the 93 patients, 75 (81%) received adjuvant chemotherapy and 41 (44%) received adjuvant radiotherapy. The majority of TNBC patients either received an anthracycline-based chemotherapy regimen with a taxane (26%) or with fluorouracil (27%) which was not significantly different between the two races (63% of AA patients versus 53% of Caucasian patients, *P* = 0.06)(not shown).

### Overall survival

**Figure 1:**
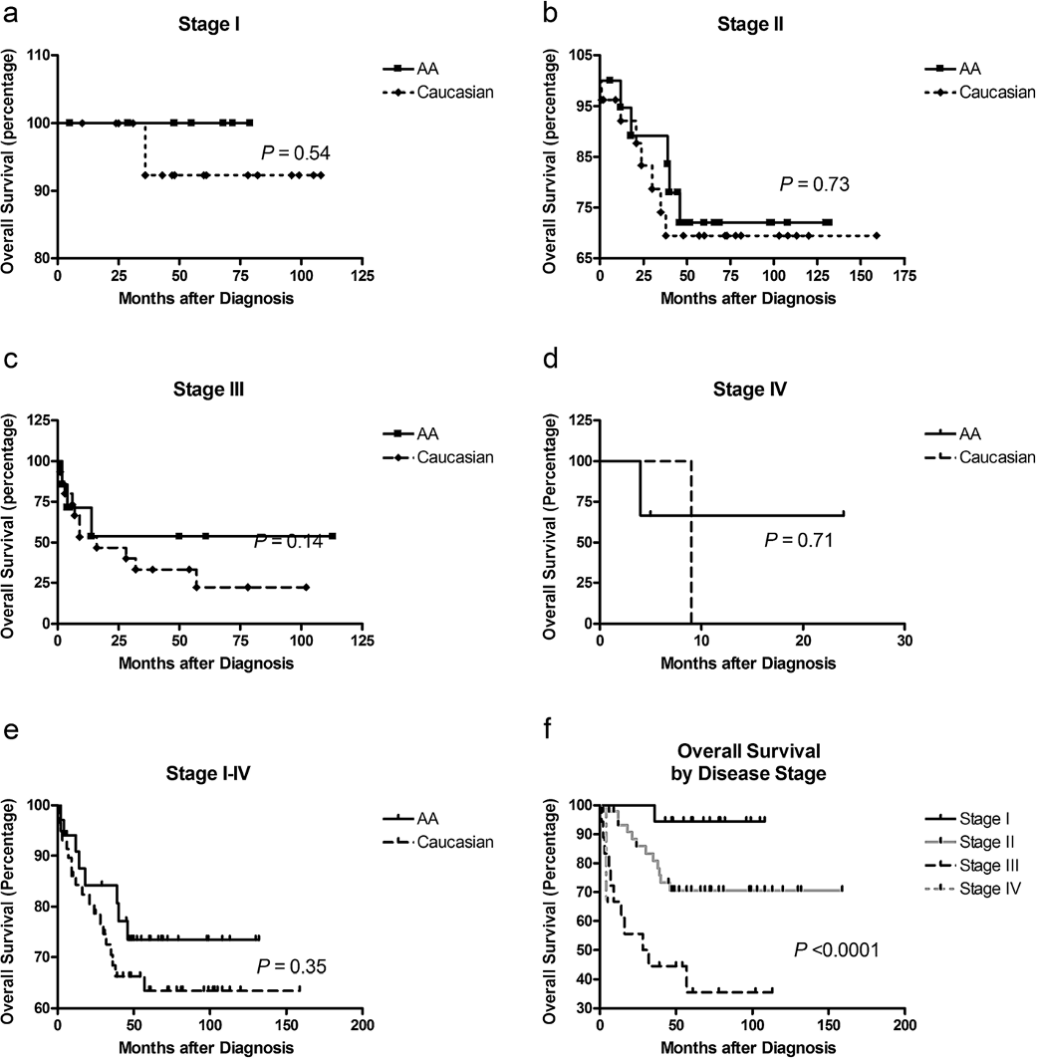
**Overall survival (OS) months after diagnosis by race and/or stage of TNBC. a)** OS by race in stage I TNBC, **b)** OS by race in stage II TNBC, **c)** OS by race in stage III TNBC **d)** OS by race in stage IV TNBC **e)** OS by race in stage I-IV TNBC **f)** OS by stage of TNBC.

Of the 93 TNBC patients, 80 patients (86%) were either alive or lost to follow-up at the end of our study. Figure 1a-f illustrate the Kaplan-Meier survival curves with the corresponding log-rank *P*-values for OS by race and stage of breast disease. Of women diagnosed with stage I breast cancer, the OS rates for AAs was 100% compared to Caucasians at 94% (95% CI, 0.003 to 19; *P* = 0.54) (Figure 1a). For women with stage II breast cancer, OS for AA women was 85% and for Caucasian women was 86% (HR = 0.8; 95% CI, 0.3 to 2.6; *P* = 0.73) (Figure 1b). For advanced stages (stage III and IV), survival for AA women were 78% and 40% for Caucasian women (HR = 0.6; 95% CI 0.2 to 1.98; *P* = 0.43) (Figure 1c and d). Collectively, our results demonstrate that differences in OS due to race and overall stage of breast disease between the two races are not statistically significant (Figure 1e and f).

### Cancer recurrence and mortality

**Figure 2:**
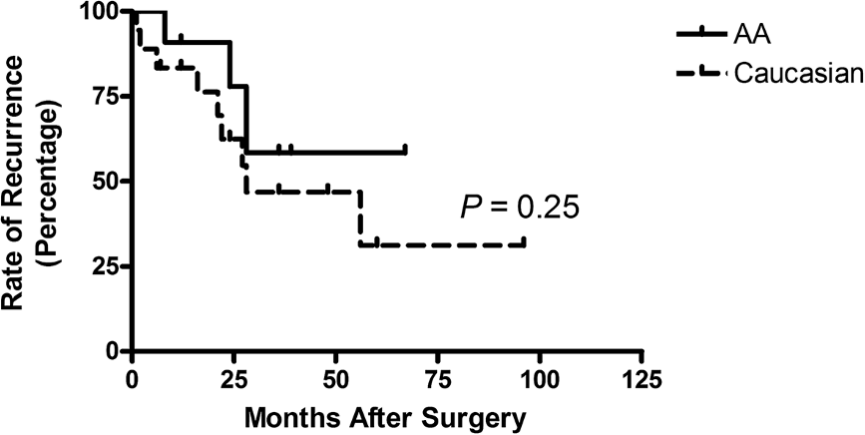
**Rate of breast cancer recurrence months after surgery by race.**

Cancer recurrence rates was determined by stage and race among AA and Caucasian TNBC patients following surgery from the year 2000 to 2010 (Table 2). Of a total of 23 stage I TNBC patients, 2 AA (9%) and 4 Caucasian patients (17%) experienced a recurrence. The majority of TNBC patients who experienced a recurrence from the disease had stage II TNBC (n = 17). Of a total of 48 stage II TNBC patients, 7 AAs (15%) and 10 Caucasians (21%) experienced cancer recurrence. Of the 18 stages III breast cancer patients, a total of 2 AA (11%) and 6 Caucasian (33%) women experienced a recurrence from an initial diagnosis of breast cancer. There was no significant difference in cancer recurrence rates between the two races (*P* = 0.25) (Figure 2).

**Table 2:** **Cancer recurrence rates by stage and race among African American (AA) and Caucasian women after surgery from 2000 to 2010**

	AA (n = 35)	Caucasian (n = 58)	AA + Caucasian (n = 93)	***P*** -value
Stage I	2	4	6	0.56
Stage II	7	10	17	0.45
Stage III	2	6	8	0.95
Stage IV	2	1	3	-
Total	13 (37%)	21 (36%)	34 (37%)	

Percentages represent the total number of patients per group divided by the total number of patients by race in the study (n) × 100.

**Figure 3:**
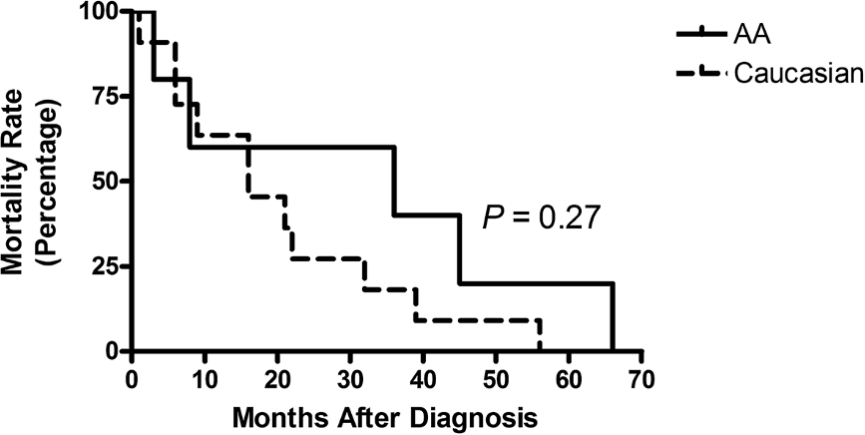
**Rate of mortality months after diagnosis by race.**

Mortality rate was also determined by stage and race among AA and Caucasian TNBC patients from the year 2000 to 2010 (Table 3). Similar to recurrence, mortality rates did not differ significantly between the two races when stage of disease was considered (*P* = 0.71) (Figure 3).

**Table 3:** **Mortality rate by stage and race among African American (AA) and Caucasian women after diagnosis during 2000 to 2010**

	AA (n = 35)	Caucasian (n = 58)	AA + Caucasian (n = 93)	***P*** -value
Stage I	0	1	1	-
Stage II	3	4	7	0.09
Stage III	1	8	9	-
Stage IV	1	1	2	-
Total	5 (14%)	14 (24%)	19 (20%)	

Percentages represent the total number of patients per group divided by the total number of patients by race in the study (n) × 100.

### Cox proportional hazard modeling

Table 4 illustrates patient variables that were fitted to the Cox proportional hazard model to determine survival outcome. When comparing AA versus Caucasian TNBC patients and controlling for patient and tumor characteristics, race was not significantly associated with OS (HR = 1.24; 95% CI, 0.32 to 5.08; *P* = 0.74). Patients who achieved a pCR had significantly decreased hazard of death (HR = 0.16; 95% CI, 0.06 to 0.41; *P* = 0.0002) compared to patients who did not achieve a pCR. Similarly, patients who had earlier stage of TNBC had significantly better OS (HR = 0.10; 95%CI, 0.01 to 0.13; *P* < 0.0001).

**Table 4:** **Multiple variable cox proportional hazard models**

Overall survival			
Variable	HR	95% CI	***P***
Caucasian vs. AA	1.24	0.32 to 5.08	0.74
pCR: yes vs. no	0.16	0.06 to 0.41	0.0002
Clinical Stage: I/II vs. III/IV	0.10	0.01 to 0.13	<0.0001
Premenopausal vs. Postmenopausal	1.30	0.44 to 3.93	0.24
XRT vs. No XRT	0.47	0.17 to 1.42	0.19
Chemo v. No Chemo	0.81	0.20 to 3.36	0.78

*Abbreviations*: *pCR* pathologic complete response, *XRT* radiotherapy; *Chemo* chemotherapy.

## Discussion

Breast cancer is the most prevalent malignancy and the second leading cause of cancer death among women in the United-States (DeSantis et al. 2011). It is well documented that breast cancer incidence is higher among Caucasian women, compared to other ethnic groups (DeSantis et al. 2011). However, several studies have noted an increased incidence, late stage at diagnosis, and poorer survival outcome among young, AA women of childbearing age diagnosed with TNBC (Stead et al. 2009; Anderson et al. 2008; Tawfik et al. 2010). TNBC is characterized by a lack of ER, PR, and HER-2/neu receptors and an aggressive, basal-like phenotype that is most prevalent among AA women (Ray & Polit 2010). The increased incidence and mortality rates in AA vs. Caucasian TNBC patients may be multifactorial, including advanced stage of disease at the time of diagnosis (Weir et al. 2003), less prompt access to medical care (Shavers & Brown 2002), and socioeconomic factors (Brawley 2002).

There are limited studies that address the issue of race and survival for women with TNBC. In one large cohort study, consisting of 6370 women identified as having triple-negative breast cancer, women with triple-negative breast cancers were significantly more likely to be non-Hispanic black (odds ratio = 1.77), under the age of 40 (odds ratio [OR], 1.53), and had worse survival, with a 5-year relative survival of only 14% (Bauer et al. 2007). Similarly, a study by Stead et al. reported that the odds of having a triple negative tumor were 3-fold higher (HR = 5.5, 95% CI 1.6, *P* = 0.0001) in black compared with white women (Stead et al. 2009). Contrary to these studies, our study demonstrates for the first time that there is no significant difference in OS between AA and Caucasian TNBC patients treated at the UAMS Women’s Oncology Clinic in Arkansas (HR = 1.24; 95% CI, 0.32 to 5.08; *P* = 0.74). An interesting observation in our cohort is the age and menopausal status of our patients, with 65% being postmenopausal and over half of the patients being age 50 or older at diagnosis, which is contrary to the previously observed association between TNBC and younger age and premenopausal status (Anderson et al. 2008; Ray & Polit 2010). In addition, we show that there is no significant difference in the likelihood of receiving adjuvant chemotherapy and radiotherapy by race. Due to the retrospective nature of our study and the limitation of our small patient population size, which may account for the non-significant differences between race and survival outcomes, no power calculations were performed. However, our cohort, like MD Anderson’s, represents a geographically uniform group of TNBC patients with similar socioeconomic and cultural risks treated with a similar multidisciplinary approach at UAMS in Little Rock, Arkansas. In that study reported by Dawood et al., 470 patients with TNBC were treated with primary systemic chemotherapy at the MD Anderson Cancer Center. Their results demonstrated that race does not significantly affect pCR rates or survival outcomes in women with TN breast cancer (Dawood et al. 2009). In addition, the authors found that the 3-year OS rate was similar between 100 black patients (68%) and 371 white/other race (62%) patients who received the same treatment conditions (*P* = 0.091). Further, RFS (HR = 1.08; 95% CI, 0.69 to 1.68; *P* = 0.747) and OS (HR = 1.08; 95% CI, 0.69 to 1.68; *P* = 0.735) was similar between the two races even after controlling for patient and tumor characteristics. If there is no significant difference between AAs and Caucasians in response to traditional cytotoxics, is it possible that a difference by race might exist in response to biologic agents? We conducted a small prospective phase II study using standard chemotherapy and bevacizumab in the neoadjuvant setting for locally advanced or operable breast cancer at UAMS. AAs had 75% pCR (9/12), whereas Whites had only 28% pCR (7/25; *P* = 0.0069), possibly in part because 100% of AA (12/12) had ductal carcinoma compared with only 64% (16/25) of Whites (*P* = 0.017). Further evaluation of this question is needed to address this issue (Makhoul et al. 2013).

In conclusion, to our knowledge, this is the only retrospective study that details TNBC trends in patients from Arkansas as it relates to race, stage of breast disease at diagnosis, recurrence and mortality, and overall survival. Our study provides supporting evidence that race is not a contributing factor on cancer recurrence, mortality or survival in AA and Caucasian TNBC patients. Future prospective studies, with participation from larger institutions that are represented by a diverse breast cancer population, will be necessary to increase statistical power and to clearly define whether or not race affects clinical outcomes in patients with TNBC.

## Abbreviations

AA: African American
OS: Overall survival
TNBC: Triple negative breast cancer.
